# Tape-Worms

**Published:** 1870-07

**Authors:** 


### TAPE-WORMS.

There is reason to believe that there may be worms in all kinds of meats, although the popular impression is that they are found only in the meat of the pig. It is now certainly known that

TRICHINIASIS, as it is called, arises from eating raw pork, and that the certain prevention is the thorough cooking of pork, ham, or bacon in all its forms.

Persons who eat dried beef are liable to become wormy, unless it is thoroughly cooked. No American ever thinks of eating any form of hog flesh without its having been first well cooked; but many eat

CHIPPED BEEF, which often has the germ of the tape-worm; this chipped beef has never been cooked, but only salted, and then dried and placed on the table as a relish; this should never be done until it has been thoroughly cooked.

It is not meant that there are young tape-worms in every plate of chipped beef; but that any piece of chipped beef is liable to have the young worms in it, and there is only one safe plan — cook all meats thoroughly. There is measly veal as well as measly pork, and every "measle" is but the encasement of a living worm, which if swallowed, without being roasted or boiled to death, will burrow in the system, and, unless killed in some other way, is liable to reproduce itself in immense numbers, or add joint to joint until it is several yards long. Tape-worms come from eating measly meat. There are no worms in healthy beef.

